# Occurrence of Diverse Antimicrobial Resistance Determinants in Genetically Unrelated Biocide Tolerant *Klebsiella pneumoniae*

**DOI:** 10.1371/journal.pone.0166730

**Published:** 2016-11-21

**Authors:** Amitabha Mondal, Manjunath Venkataramaiah, Govindan Rajamohan, Vijaya Bharathi Srinivasan

**Affiliations:** Council of Scientific Industrial Research- Institute of Microbial Technology, Sector 39 A, Chandigarh, 160036, India; University of Illinois at Chicago, UNITED STATES

## Abstract

Nosocomial infections due to *Klebsiella pneumoniae* is a significant problem in health care settings worldwide. In this study, we examined the antimicrobial susceptibility, genetic profiles and mechanisms of antibiotic resistance in *K*. *pneumoniae* isolates of Indian origin. To our knowledge this is the first report demonstrating the high prevalence of β-lactamases, aminoglycoside modifying enzymes, quinolone resistance genes besides demonstrating the involvement of active efflux in *K*. *pneumoniae* Indian isolates. This study has enabled us to correlate the phenotypic and genotypic characteristics in *K*. *pneumoniae*, providing an important base for continued monitoring and epidemiological studies of this emerging nosocomial pathogen in Indian hospitals.

## Introduction

*Klebsiella pneumoniae* is a rapidly emerging nosocomial pathogen that causes wide variety of infections from mild to severe including bacteraemia, pneumonia, meningitis, urinary tract infections (UTIs), wound infections and neonatal septicemia [1]. Infectious diseases society of America has reported this organism to be amongst top five causing major diseases and mortality in the United States [2]. According to the documented reports, *Klebsiella spp*. is responsible for 8% of total hospital acquired infections (HAIs) in the USA and Europe and is at 8^th^ position in a list of most important nosocomial pathogen in hospitals [3].A hallmark of *K*. *pneumoniae* infection is the difficulty to treat illness because of its high-level multiple intrinsic antibiotic resistance trait, which is responsible for decreased effectiveness of antibacterial therapy and thus remains as a great threat for clinicians [4].

Resistant strains of *K*. *pneumoniae* are reported in several endemic and epidemic hospital infection outbreaks [5].A study by the National Healthcare Safety Network in the USA for over a 12 month period from 2006–2007 revealed that *K*. *pneumoniae* was the causative agent for 5.8% of HAIs, and 7.7% of catheter-associated UTIs [6]. The same study showed that 21.2% of those *K*. *pneumoniae* isolates were resistant to cephalosporins and 10.1% exhibited resistance to carbapenems [6]. The emergence of aminoglycoside resistant strain was first reported in the late 1940s and in 1982, extended spectrum β-lactamase (ESBL) producing strains were identified [7]. In response to the ESBL-producing strains, carbapenem drugs were used extensively and soon carbapenemases producing strains were reported at high frequency [8]. According to CDC report in 2007, *K*. *pneumoniae* carbapenamase (KPC) producing strains are of great concern since its prevalence rises from less than 1% to 8% [8]. In another report, KPC producing *K*. *pneumoniae* was reported to have tigecycline and elevated polymyxin–B resistance [9]. Recently, New Delhi Metallo β-lactamase (NDM) producing strains were emerging globally and such strains are difficult to treat due to its pan-resistance [10]. Currently, the strains are also resistant to clinically useful drugs such as imipenem and ceftazidime [11].

Multidrug resistance in this organism has been attributed to production of modifying enzymes such as aminoglycoside modifying enzymes (AMEs), ESBLs, KPCs, NDM-1, alteration of antibiotic targets such as *gyrA* and *parC* and reduced outer membrane permeability [3, 12]. Additionally, mobile genetic elements such as plasmids and integrons quintessential elements play vital role in the development of drug resistance [13]. Another important mechanism that *K*. *pneumoniae* uses to subvert the action of antimicrobial agents is the active efflux pumps that recognize and actively export a wide variety of structurally unrelated compounds, including antibiotics and antibacterial peptides from the bacterial cell [14]. In Gram-negative bacteria including *K*. *pneumoniae*, the pumps belonging to the resistance-nodulation-division (RND) family are significantly effective in generating resistance, as they form a tripartite complex together with the periplasmic proteins belonging to the membrane-fusion-protein family and the outer membrane channels, so that drugs are pumped out directly to the external medium. The RND pumps often have wide substrate specificity [15, 16]. The first discovered member of RND family exporter in *K*. *pneumoniae* is AcrAB system, known to pump out mostly aminoglycosides, tetracycline, erythromycin, fluoroquinolones and structurally unrelated compounds [17]. In *K*. *pneumoniae* deletion of *acrAB* rendered the organism increased susceptible to quinolones and decrease in MIC of erythromycin, tetracycline, chloramphenicol, aminoglycosides, β-lactams and carbapenamase [17]. Previous study had shown the role of *kexD* in extrusion of erythromycin, novobiocin, rhodamine 6G, ethidium bromide and tetraphenylphosphonium chloride [18]. Similarly, introduction of *kdeA* into cells of hyper susceptible *E*. *coli* strain KAM32 resulted in elevated minimum inhibitory concentrations of chloramphenicol, norfloxacin, acriflavine, and ethidium bromide [19]. The efflux pumps *kpnEF* and *kpnGH* has been reported to display a role in conferring broad spectrum antimicrobial resistance previously [20, 21].

*K*. *pneumoniae* is an important cause of multidrug resistant HAIs, even in India especially in the neonatal intensive care unit (ICU) where mortality rates can reach up to 70% [22]. Jayaraman *et al* has previously reported that >60% of *K*. *pneumoniae* isolates were not only resistant to both meropenem and imipenem but also harbored *bla*_KPC_ [23]. In another study, high occurrence of ESBL in *K*. *pneumoniae* isolates was reported from South India [24]. Detection of *bla*_CTX-M-1_ and *bla*_TEM_ in *K*. *pneumoniae* isolated from pigs in north eastern India has been reported previously [25]. In a previous study, Roy *et al* has demonstrated the high prevalence of *bla*_TEM_, *bla*_SHV,_ *bla*_CTX-M_ and *bla*_QNR_ genes in pathogenic *K*. *pneumoniae* isolates collected from Kolkata India [26].

Though there exists few sporadic reports, no systematic investigation has been conducted so far that has focused on to delineate the contributions of different resistance determinant in *K*. *pneumoniae* in India in detail. The present study was conducted to elucidate the current trends in antibiotic resistance profiles and prevalence of diverse antibiotic resistance determinants in clinical isolates of *K*. *pneumoniae* collected from medical centers in India.

## Materials and Methods

### Bacteriology and clonality

*K*. *pneumoniae* strains were collected from Chennai and Chandigarh during the period 2012–2013 and were streaked on *Klebsiella* agar selective media from Hi Media. Based on the growth of culture on selective agar, the isolates were confirmed to be *K*. *pneumoniae*. To determine the lineage to which these strains belong, we standardized BOX-PCR and ERIC-PCR in this study to determine the clonality among the isolates [27].

### Antibiotic susceptibility testing

Strains in this study were examined for resistance to imipenem: IMP (10 μg/ml), ertapenem: ETP (10 μg/ml), ceftriaxone: CTR (30 μg/ml), ceftazidime: CAZ (30 μg/ml), cefepime: FEP (30 μg/ml), nalidixic acid: NAL (30 μg/ml), norfloxacin: NOR (10 μg/ml) enrofloxacin: ENX (10 μg/ml), ciprofloxacin: CIP (5 μg/ml), gentamicin: GEN (10 μg/ml) kanamycin: KAN (30 μg/ml), streptomycin: STR (10 μg/ml), tobramycin: TOB (10 μg/ml), spectinomycin: SPT (100 μg/ml), azithromycin: AZM (15 μg/ml), erythromycin: ERY (15 μg/ml), tetracycline: TET (30 μg/ml), rifampicin: RIF (5 μg/ml), trimethoprim: TMP (5 μg/ml), linezolid: LZD (5 μg/ml), doxycycline: DOX (30μg/ml), minocycline: MIN (30μg/ml), chloramphenicol: CHL (30 μg/ml), colistin: CST (10 μg/ml) and tigecycline: TGC (30μg/ml) by using commercially available discs (HiMedia Laboratories, India)as described previously and data was analyzed according to the interpretation criteria recommended by Clinical and Laboratory Standards Institute (CLSI) [28].

### Determination of Minimum Inhibitory Concentration (MIC)

1. **Concentrations of antibiotics tested.** MIC was determined for the clinical isolates by the agar diffusion method. Luria-Bertani (LB) plates containing various concentrations of the drug were prepared and the overnight cultures were freshly inoculated to achieve the OD_600nm_ of 0.1 and culture was then spotted on each LB plate having different concentration of the antimicrobials. The plates were then incubated at 37°C for 16 h and then growth was monitored [29, 30]. In this study we used 18 antibiotics from different classes viz. β-lactams (ampicillin, carbenicillin, ceftazidime, cefotaxime), aminoglycosides (amikacin, gentamicin, kanamycin, neomycin, streptomycin), quinolones (ciprofloxacin, nalidixic acid, norfloxacin), and others (erythromycin, chloramphenicol, polymyxin B, rifampicin, tetracycline and trimethoprim) at different concentrations (0.5 μg/ml, 4 μg/ml, 16 μg/ml, 64 μg/ml, 256 μg/ml and 1024 μg/ml). We calculated the MIC from inhibition of growth in plates with antibiotics with respect to control plate having no added supplement in it.
2. **Concentrations of different compounds tested.** We checked for MIC of sodium dodecyl sulphate (SDS) (detergent), deoxycholate (bile salt), indole, sodium salicylate, sodium tungstate, acridine orange, acriflavine and rhodamine G (dyes, which are also known as substrates for efflux pump). The varied concentrations used for the testing was 16 μg/ml, 64 μg/ml, 256 μg/ml, 1024 μg/ml, 4096 μg/ml and 16384 μg/ml for SDS, deoxycholate, acridine orange, acriflavine and rhodamine G and 0.5 mM, 1 mM, 2.5 mM, 5 mM, 10 mM and 25 mM for indole, sodium salicylate and sodium tungstate respectively.
3. **Concentrations of hospital based disinfectants tested.** Similarly, we checked the MIC of the strains for biocides (benzalkonium chloride, chlorhexidine & triclosan) which are regularly used as component of disinfection and cleansing solution and also some commercially available disinfectants (Domex and Lyzol) which are regularly used in hospital set up for cleaning proposes. For benzalkonium chloride and chlorhexidine, concentrations tested were 3.2 μg/ml, 6.4 μg/ml, 12.8 μg/ml, 25.6 μg/ml, 51.2 μg/ml and 102.4 μg/ml and for triclosan the values tested were 0.001 μg/ml, 0.005 μg/ml, 0.01 μg/ml, 0.05 μg/ml, 0.1 μg/ml and 0.5 μg/ml and for Domex & Lyzol we used plates with 0.1%, 0.2%, 0.4%, 0.8%, 1.2% and 1.6%. The experiments were repeated three times.

### Bacterial transformation and plating on selective media

Plasmid DNA was extracted from the isolates by a method described previously [31], except that for plasmid isolation, 0.75ml of culture and twice the suggested volume of all three solutions were used. *E*. *coli* JM109 electro competent cells, prepared according to the manufacturer’s recommendations (Bio-Rad Laboratories, Richmond, Calif.), were transformed with 30 ng of plasmid preparations as described before [32].

### Genomic DNA isolation, PCR amplification, sequence analysis

Presence of resistance determinant genes in the chromosome of the clinical isolates were detected by PCR based approach. The genomic DNA from all the isolates was extracted using the CTAB method and was used as a template all the PCRs performed. Clonal relatedness was determined by ERIC-PCR using primers ERIC-1R: ATGTAAGCTCCTGGGGATTCAC and ERIC-2: AAGTAAGTGACTGGGGTGAGCG and products were analyzed on gel. Resistance genes were determined by using specific sets of gene specific primers [33–39]. We checked for presence of ESBL genes by performing PCR for *bla*_SHV_, *bla*_VEB_ and *bla*_GES;_ metallo β lactamases genes like *bla*_SIM_, *bla*_VIM_, *bla*_GIM_, *bla*_IMP_, and *bla*_SPM;_ genes investigated also included carbapenemase genes *bla*_OXA-23,_ *bla*_OXA-24_, *bla*_OXA-58_ and *bla*_OXA-69_. Presence of genes for conferring resistance to aminoglycosides like *aadA1*, *aphA3*, *aphA6*, *rmtB*, *rmtC*, *rmtD*, *strA*, *strB* were also determined. We also checked for the presence of quinolone resistance genes *qnrA qnrB*, *qnrS* and some other genes like *catIII*, *catB2*, *catB3*, *cmlB* and CusABC efflux pump (epF: 5’–GATATGGATCTGGTGCCGAAATA– 3’ and epR: 5’ ACTGCGAGGCGTCTTTAATC– 3’). Elution of the amplicons was done as described previously. Sequencing was carried out by following the manufacturer’s instructions (Big Dye Terminator kit; Applied Biosystems) with an ABI Prism 310 instrument. The identities of the sequences were established through a database search by using the BLAST program [40, 41].

### Growth inhibition assay to elucidate the active efflux activity

The assays were performed as described previously [29]. Cultures in the mid-exponential phase were inoculated into LB broth containing antimicrobials either alone or with the efflux pump inhibitors and the growth profile of the cultures at 37°C and was analyzed by measuring the absorbance at OD600_nm_ periodically at a regular interval thereafter using Synergy H1 Hybrid microplate reader (BioTek Instruments Inc., Winooski VT). In this study, we used efflux based substrates antibiotics {kanamycin 16 μg/ml}, {acriflavine 8µg/ml}, and disinfectants such as {benzalkonium chloride 3.2µg/ml} as substrates. The 2, 4-dinitrophenol (DNP), carbonyl cyanide 3- chloro phenyl hydrazone (CCCP), verampamil (VER) and reserpine (RES) were used as efflux pump inhibitors {2.5 μg/ml} in this study. We analysed efflux activity as the difference in growth profile of the strains with the presence and absence of drug along with presence and absence of different inhibitors in independent experiments.

### Analysis of outer membrane protein profiles

Outer membrane proteins were purified by the method as described previously [42], with some slight modification. Cells were harvested by centrifugation (6,000 × g for 15 min) and were suspended in 50 mM potassium phosphate buffer (pH 7.2) and sonicated for 5 mins. The cells were crushed by sonication and the unbroken cells were removed by centrifugation (10,000 × g for 10 min). The crude envelope fraction was collected from the supernatant by centrifugation at 105,000 x g for 1 h at 4°C. The pellet containing the crude envelope fraction was treated with 0.5% (wt/vol) sarkosyl (Sigma) solution for 30 mins to selectively solubilize the inner membrane part. The insoluble outer membrane fraction (OMP) was recovered as pellet by centrifugation at 105,000 x g for 1 h at 4°C. The pellet was resuspended and stored at -20°C until used. Protein contents of membrane preparations were determined by the method of bicinchoninic acid (BCA) method (Pierce BCA protein assay kit) with bovine serum albumin (BSA) (Sigma) as standard.

### Estimation of biofilm formation

In this study, the overnight cultures were freshly inoculated to achieve an initial OD_600nm_ of 0.1, and then incubated at 37°C for 16 h and were observed for the growth and measured as OD_600nm_ then the culture were discarded and washed carefully without disturbing possible biofilm formation along the wall. Tubes were then filled with 0.1% crystal violet and incubated for 30 min. Later stain was discarded, washed and dried. Now biofilm was dissolved using 1 ml 33% acetic acid and OD was measured at 570nm [29]. Biofilm formation was calculated as the ratio of OD_570nm_/OD_600nm._

### Oxidative stress tolerance assay

We performed oxidative disc diffusion assay to know the oxidative stress tolerance of the clinical isolates. Hydrogen peroxide (H_2_O_2_) was used to generate oxidative stress in our current study. The test cultures were grown overnight and then diluted to make OD_600nm_ equivalent to 0.1. The cultures were spread on the LB plates and Whatman filter paper discs soaked in different concentrations of H_2_O_2_ (0%, 0.1%, 1%, 3%, or 10%) are placed at appropriate distances from one another and incubated at 37°C for 16 h. After incubation, the zone of inhibition around every H_2_O_2_ disc was measured and compared [30].

### Precipitation test and Motility Assay

For the precipitation test, 1 ml cultures were centrifuged at 4000g for 2 mins and left in standing condition for 15 mins, later visually tested for mucoidy or no -mucoidy supernatant and dense pellet. The motility assays were done with representative strains where LB grown *K*. *pneumoniae* cultures (OD_600nm_ = 1.0) were inoculated with a toothpick on LB agar plates with 0.25%, 0.45% and 0.7% agar and incubated for 12 hrs at 37°C. In this growth medium, bacteria can swim through the soft agar and produce a halo. The diameter of the halo is a measure of the ability to swarming motility. And some bacteria use to have twitching motility which is also detected using staining of bacterial cells in lower bottom of media as zone of twitching motility [30].

## Results

### Clonal relation among of Indian *K*. *pneumoniae* isolates

**Fig 1:**
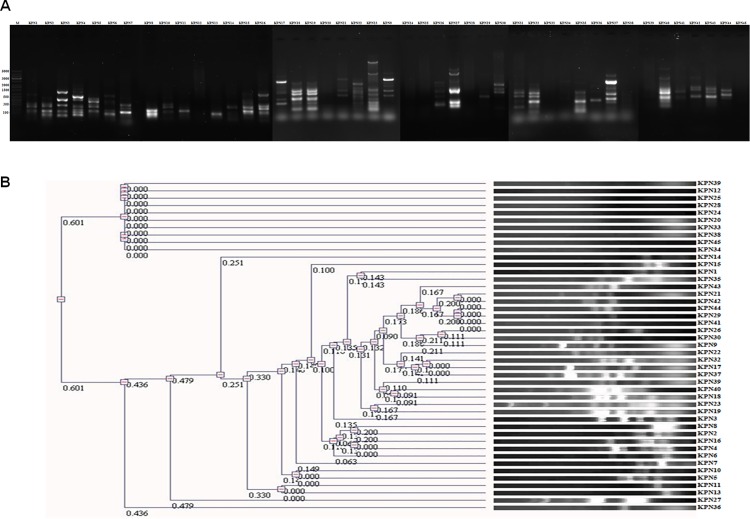
Clonal relationship among the *K*. *pneumoniae* strains from medical centre in India. Clonal relationship among the *K*. *pneumoniae* strains obtained from medical centers of India was established through PCR approach using specific primers for ERIC-PCR which showed a wide variety of clonal types among the isolates, while few strains had similar band pattern. (A) DNA gel picture of amplicon obtained by ERIC PCR of the *K*. *pneumoniae* isolates.(B) Dendogram formed by neighbor joining method in UVP software from the band pattern of the *K*. *pneumoniae* strains obtained by ERIC PCR showing 12 different clusters.

*K*. *pneumoniae* strains were collected from Chennai and Chandigarh for a period of 2012–2013 and identified isolates as *K*. *pneumoniae* strains. The strains were collected from medical centre as kind gifts. To understand the clonal relationship among the isolates we used ERIC PCR (Fig 1A) and BOX PCR based (data not shown) approaches. Based on the dendogram the isolates used in this study were diverse, clonally distinct and could broadly classified into 12 different clusters (Fig 1B).

### Drug resistance profiles of Indian *K*. *pneumoniae* isolates

**Fig 2:**
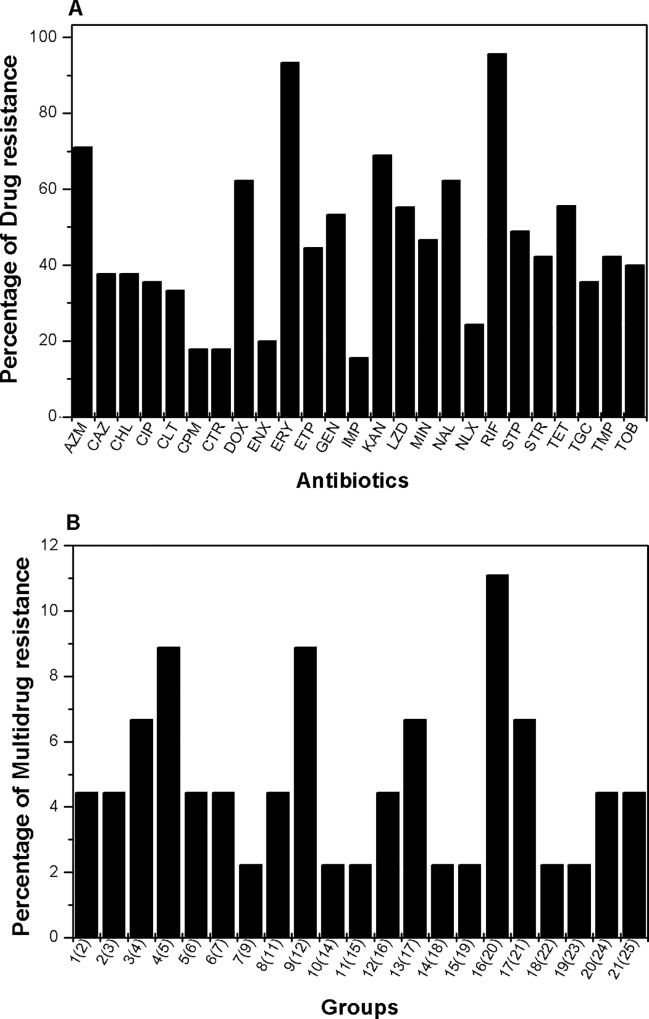
Antibiotic susceptibility testing of *K*. *pneumoniae* strains from medical centre in India. (A) Graphical presentation of percentage of isolates exhibiting resistance to respective antibiotics used in this study. To determine the percentage of drug resistance, disc diffusion assay was performed using AZM: azithromycin, CAZ: ceftazidime, CHL: chloramphenicol, CIP: ciprofloxacin, CST: colistin, CTR: ceftriaxone, DOX: doxycycline, ENX: norfloxacin, ERY: erythromycin, ETP: ertapenem, FEP: cefepime, GEN: gentamicin, IMP: imipenem, KAN: kanamycin, LZD: linezolid, MIN: minocycline, NAL: nalidixic acid, NOR: norfloxacin, RIF: rifampicin, SPT: spectinomycin, STR: streptomycin, TET: tetracycline, TGC: tigecycline, TMP: trimethoprim, TOB: tobramycin, and analyzed as per standard CLSI standards. (B) The *K*. *pneumoniae* strains showed resistance toward multiple antibiotics, we grouped the pool of isolates as per the number of antibiotics they were showing resistance to. Here percentages of isolates belonging to different groups with different number of antibiotic resistance are shown where the respective number of antibiotics is mentioned within bracket with each group.

Antibiotic susceptibility testing was performed by Kirby-Bauer assay and the antibiogram of the clinical isolates tested were prepared to know the resistance pattern. The isolates were found resistant to different antibiotics; 95% of the isolates were resistance to rifampicin, 93% of isolates were found resistant to erythromycin, 68% to kanamycin, 62% of isolates were resistant to nalidixic acid, 71% of strains to azithromycin, 55% of strains to tetracycline, and 44% of strains were resistant to ertapenem. Interestingly 35% of isolates were resistant to tigecycline (glycylcycline group of antibiotics) which is the current choice of antibiotic. We also found resistance against imipenem (15%), ceftriaxone (17%), ceftazidime (37%), cefepime (17%), gentamicin (53%), streptomycin (42%), tobramycin (40%), spectinomycin (48%), norfloxacin (24%), enrofloxacin (20%), ciprofloxacin: (35%), trimethoprim (42%), linezolid (55%), doxycycline (62%), minocycline (46%), chloramphenicol (37%) and colistin (33%) in our collection of isolates (Fig 2A, Table 1).

**Table 1:** Antibiotic susceptibility profiles of *K*. *pneumoniae* isolates.

Strains	Antibiotics																									
	AZM	CAZ	CHL	CIP	CST	CRO	DOX	ENR	ERY	ETP	FEP	GEN	IPM	KAN	LVX	LZD	MIN	NAL	NOR	RIF	SPT	STR	TET	TGC	TMP	TOB
**KPN1**	+	-	-	-	+	-	+	-	+	-	-	+	-	+	-	+	+	+	+	+	+	+	+	+	-	+
**KPN2**		+	+	+	+	+	+	+	+	+	+	+	+	+	-	+	+	+	+	+	+	+	+	+	+	+
**KPN3**	+	+	+	+	+	+	+	+	+	+	+	+	-	+	-	+	+	+	+	+	+	+	+	+	+	+
**KPN4**	+	+	+	-	+	-	+	+	+	-	+	+	-	+	-	+	+	+	-	+	+	+	+	+	+	+
**KPN5**	+	+	+	-	+	-	+	-	+	+	-	+	-	+	-	+	+	+	+	+	+	+	+	+	+	+
**KPN6**	-	+	+	-	+	+	-	-	-	+	+	+	-	+	-	+	-	-	-	+	+	-	-	-	+	-
**KPN7**	+	+	+	+	+	-	+	-	+	+	-	+	-	+	-	+	+	+	+	+	+	+	+	+	+	+
**KPN8**	+	+	+	-	+	-	+	-	+	+	-	+	-	+	-	+	+	-	-	+	+	+	+	+	-	+
**KPN9**	+	+	+	+	+	+	+	+	+	+	+	+	+	+	-	+	+	+	+	+	+	+	+	+	+	+
**KPN10**	+	+	+	-	+	-	+	+	+	+	+	+	-	+	-	+	+	+	+	+	+	+	+	+	+	+
**KPN11**	+	-	-	+	+	-	+	+	+	+	-	+	-	+	-	+	+	+	+	+	+	+	+	+	+	+
**KPN12**	+	-	+	-	+	+	+	-	+	+	-	+	-	+	-	+	+	+	-	+	+	+	+	+	+	-
**KPN13**	+	+	-	-	+	-	+	+	+	+	-	+	+	+	-	+	+	+	+	+	+	+	+	+	+	+
**KPN14**	+	+	+	+	+	+	+	+	+	+	+	+	-	+	-	+	+	+	+	+	+	+	+	+	+	+
**KPN15**	+	+	-	+	+	+	+	+	+	+	+	+	-	+	-	+	+	+	+	+	+	+	+	+	+	+
**KPN16**	-	-	-	-	-	-	-	-	+	-	-	-	-	-	+	-	-	-	-	+	-	-	-	-	-	-
**KPN17**	-	-	-	-	+	-	-	-	+	-	-	-	-	+	+	-	-	-	-	+	-	-	-	-	-	-
**KPN18**	+	-	-	+	+	-	+	+	+	-	-	-	-	+	+	-	+	+	+	+	+	-	+	+	+	-
**KPN19**	+	-	-	+	-	-	+	+	+	-	-	-	-	-	+	-	+	+	+	+	-	-	+	-	-	-
**KPN20**	-	+	-	+	-	+	+	+	+	-	+	-	-	+	+	-	+	+	+	+	-	-	+	-	-	-
**KPN21**	-	-	-	-	+	-	+	-	+	-	-	-	-	-	+	-	-	-	-	+	-	-	+	-	-	-
**KPN22**	+	-	+	-	+	+	-	+	+	+	-	+	-	+		-	+	+	+	-	+	-	+	-	-	+
**KPN23**	+	-	-	-	+	-	+	-	+	-	-	-	-	-	+	-	+	+	-	+	-	-	+	+	+	-
**KPN24**	+	+	+	+	-	+	+	+	+	+	+	+	+	+	+	-	-	+	+	+	+	-	-	-	+	+
**KPN25**	-	-	+	-	-	+	-	-	-	+	-	+	+	+	+	-	-	-	-	+	+	+	-	-	+	+
**KPN26**	-	-	-	-	-	-	-	-	+	-	-	-	-	-	+	-	-	-	-	+	-	-	-	-	-	-
**KPN27**	-	-	-	-	+	-	-	-	+	-	-	-	-	-	+	-	-	-	-	+	-	-	-	-	-	-
**KPN28**	+	-	-	+	+	+	+	+	+	-	-	+	-	+	+	-	+	+	+	+	-	-	+	-	+	+
**KPN29**	+	+	+	+	+	+	+	+	+	+	+	+	+	+	+	-	-	+	+	+	-	-	-	-	+	-
**KPN30**	+	+	+	-	+	+	+	+	+	+	+	+	+	+	+	-	+	+	+	+	-	-	-	-	+	+
**KPN31**	-	-	-	-	-	-	-	-	+	-	-	-	-	+	-	-	-	-	-	+	-	-	-	-	-	-
**KPN32**	+	+	-	-	-	-	-	-	+	-	-	-	-	-	-	-	-	+	-	+	-	-	-	-	-	-
**KPN33**	+	-	-	-	-	-	+	-	+	-	+	-	-	+	-	-	-	+	+	+	-	+	+	-	-	-
**KPN34**	+	-	-	+	-	-	+	-	+	-	-	+	-	-	-	-	-	+	+	+	+	-	+	-	-	-
**KPN35**	+	-	-	-	-	-	-	-	+	-	-	-	-	-	-	-	-	-	-	+	-	-	-	-	-	-
**KPN36**	-	-	-	-	-	-	-	-	+	-	-	-	-	-	-	-	-	-	-	-	-	-	-	-	-	-
**KPN37**	+	-	+	+	-	+	+	-	+	-	+	+	-	+	-	-	-	+	+	+	+	+	+	-	-	-
**KPN38**	+	-	-	-	-	-	-	-	+	-	-	-	-	+	-	-	-	-	-	+	-	-	-	-	-	-
**KPN39**	+	-	-	-	-	-	-	-	+	-	-	-	-	-	-	-	-	-	-	+	-	-	-	-	-	-
**KPN40**	+	-	-	-	-	-	-	-	+	-	-	-	-	-	-	-	-	-	-	+	-	-	-	-	-	-
**KPN41**	+	-	+	+	-	+	-	-	+	+	+	+	-	+	-	-	-	+	+	+	+	-	-	-	-	-
**KPN42**	-	-	-	-	-	-	-	-	+	-	-	-	-	-	-	-	-	-	-	+	-	-	-	-	-	-
**KPN43**	+	-	-	-	-	+	-	-	+	-	-	-	-	+	-	-	-	-	-	+	-	+	-	-	-	-
**KPN44**	-	-	-	-	-	-	+	-	+	-	-	-	-	+	-	-	-	-	-	+	-	+	+	-	-	-
**KPN45**	-	-	-	-	-	-	+	-	+	+	-	-	-	-	-	-	-	+	-	+	-	-	-	-	-	-

### Multidrug resistance profiles of Indian *K*. *pneumoniae* isolates

We analyzed the MDR pattern of isolates and found that many were multidrug resistant. We classified the isolates into groups based on their antibiotic resistance profile (Fig 2B, Table 1). Interestingly 4.4% of isolates showed resistance to all the 25 antibiotics used and alarmingly high percentage (31%) of isolates were resistant to 20 or more antibiotics.

### Minimum Inhibitory Concentration (MIC) for antibiotics

To know the extent of resistance, MIC was determined for 18 antibiotics from different classes; based on the obtained data the isolates were grouped into highly resistant, intermediate and low level resistant. For carbenicillin, we found 43% of isolates were having high MIC values from 256 μg/ml to >1024 μg/ml and 46% of isolates had values ranging from 16 μg/ml to 64 μg/ml. For another β-lactam antibiotic ceftriaxone; 43% of isolates had high MIC values ranging from 256 μg/ml to >1024 μg/ml and 53% of isolates had lower range between 0.5 μg/ml to 4 μg/ml. For ceftazidime, we found 60% of strains being susceptible to MIC of 4 μg/ml or less and 16% of isolates had MIC between 256 μg/ml to >1024 μg/ml and another 23% have an intermediate range from 16 μg/ml to 64 μg/ml.

For amikacin, we found 20% of the strains were having MIC values >256 μg/ml while 30% of them had MIC values in range of 16 μg/ml to 64 μg/ml and remaining 50% having value of 4 μg/ml or less. For gentamicin, 34% of the strains had MIC values >256 μg/ml while 65% of them had MIC values of 4 μg/ml or less. For kanamycin, 26% of strains had high MIC values of >1024 μg/ml, another 26% of isolates had intermediate MIC values of 16 μg/ml to 64 μg/ml while 45% of them had lower MIC values between 4 μg/ml and 0.5 μg/ml. For another aminoglycoside neomycin 75% of isolates were susceptible having MIC values lower than 4 μg/ml and 13% had high MIC values of 256 μg/ml. For streptomycin, 13% of them showed high MIC values (256 μg/ml to >1024 μg/ml) and 48% had MIC values of 16 μg/ml to 64 μg/ml and 37% displayed MIC values of 4 μg/ml or less.

In this study, 20% of isolates had high MIC values for ciprofloxacin, from 256 μg/ml to >1024 μg/ml, another 20% had intermediate range from 16 μg/ml to 64 μg/ml and 60% of them had MIC values in a lower range of 4 μg/ml or less. For nalidixic acid, a synthetic drug 56% of isolates was highly resistant with MIC values 256 μg/ml to >1024 μg/ml and 36% of them had MIC values in a lower range of 4 μg/ml or less. For norfloxacin 20% isolate had MIC from 256 μg/ml to >1024 μg/ml and another 20% have in intermediate range from 16 μg/ml to 64 μg/ml and 60% had MIC values in a lower range of 4 μg/ml or less.

For chloramphenicol, 73% of isolates were susceptible with MIC values in range of 4 μg/ml or less and 13% of them had MIC values of 256 μg/ml to >1024 μg/ml. Most of the isolates (86%) showed high MIC from 256 μg/ml to >1024 μg/ml, another 6% have in intermediate range from 16 μg/ml to 64 μg/ml and 6% of them had MIC values in a lower range of 4 μg/ml or less for erythromycin. We found most (96%) of strains were susceptible to polymyxin-B having MIC of 4 μg/ml or less but 3% of strains have MIC value of 256 μg/ml.

**Fig 3:**
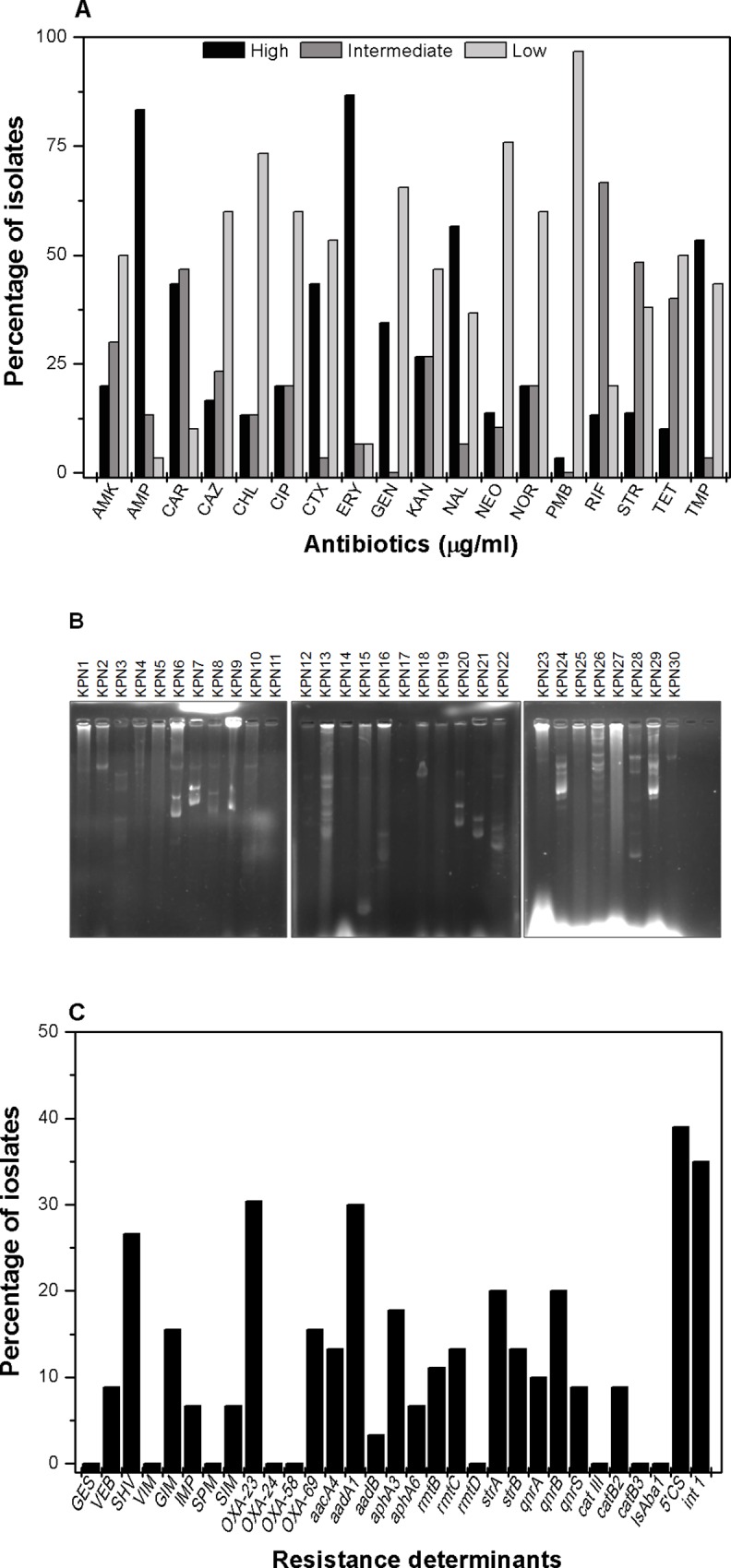
Phenotypic and genotypic characterization of *K*. *pneumoniae* strains from medical centre in India. (A) Graphical presentation of percentage of isolates with different level of resistance with different antibiotics. In this study we grouped the isolates in three different resistance level for each antibiotic *viz*. strains with high MIC values (256 μg/ml to >1024 μg/ml) are grouped as highly resistant, intermediate MIC value (16 μg/ml to 64 μg/ml) as intermediately resistant and low MIC value (4 μg/ml or less) as sensitive. For this study, we used the following antibiotics-AMP: ampicillin, CAR: carbenicillin, CTR: ceftriaxone, CAZ: ceftazidime, AMK: amikacin, GEN: gentamicin, KAN: kanamycin NEO: neomycin, STR: streptomycin CIP: ciprofloxacin, NAL: nalidixic acid, NOR: norfloxacin, CHL: chloramphenicol, ERY: erythromycin, PMB: polymyxin B, RIF: rifampicin, TET: tetracycline, TMP: trimethoprim. The bar graph represents the mean of three independent experiments. (B) DNA gel picture of *K*. *pneumoniae* plasmids isolated by alkaline lysis method from respective strains showed multiple plasmids of different size. The MDR strains carried multiple plasmids which indicated plasmids might play role in drug resistance. (C) The prevalence to different resistance determinants in the *K*. *pneumoniae* isolates are shown here. In this study, we found high prevalence of β-lactamases (*bla*_*GES*,_ *bla*_*VEB*,_ *bla*_SHV,_ *bla*_VIM,_ *bla*_GIM,_ *bla*_IMP,_ *bla*_*SPM*,_ *bla*_SIM,_ *bla*_*oxa 23*,_ *bla*_*oxa 24*,_ *bla*_*oxa 24*,_ *bla*_*oxa 58*,_ *bla*_*OXA69*_)_;_ aminoglycosidases(*aacA4*, *aadA1*, *aadB*, *aphA3*, *aphA6*, *rmtB*, *rmtC*, *rmtD*, *strA*, *strB)* and quinolone resistance genes *qnrA*, *qnrB*, *qnrS*, including *cat III*, *catB2*, *catB3*, IsAba1, 5' CS of Class 1 integrons.

For rifampicin we found 13% of strains have high MIC value of 256 μg/ml and another 66% have in intermediate range from 16 μg/ml to 64 μg/ml and 20% of them had MIC values in a lower range of 4 μg/ml or less. For tetracycline, 10% of the isolates were resistant to tetracycline displaying MIC values of 16 μg/ml to 64 μg/ml and 40% of isolates shown MIC values of 4 μg/ml while 50% had MIC values in low range of 0.5 μg/ml or less. For trimethoprim, more than half (53%) of the isolates showed high MIC from 256 μg/ml to >1024 μg/ml and 43% had MIC values in a lower range of 4 μg/ml or less and rest (3%) have MIC values of 16 μg/ml (Fig 3A).

### Role of plasmids in Indian *K*. *pneumoniae* isolates

Plasmids are extra chromosomal genetic elements capable of self replication and are easily transmissible. Plasmids are known to carry genes required for sustaining in adverse conditions and also genes responsible for various drug resistance. Clinical isolates were studied for the presence of plasmid. The 63% of strains were found to have multiple plasmids of various sizes (Fig 3B).Upon transforming the plasmids in *E*. *coli* JM109 electro competent cells, we found transformants on ampicillin, streptomycin, tetracycline containing antibiotic plates; work is in progress to know their precise contribution in conferring antimicrobial resistance in *K*. *pneumoniae* from Indian scenario.

### Diverse antibiotic resistance determinants in *K*. *pneumoniae*

With the precise information about the antibiotic resistance profile of these strains it was necessary to know the resistance genes present in the genome of the pathogen. Therefore the genomic DNA was isolated and gene specific primers were used to amplify the respective genes.

- The β-lactamases are known as a group of enzymes having the ability to hydrolyze β-lactam antibiotics thereby conferring resistance to the micro organisms. In our study, *bla*_SHV_ gene was present in 26%, *bla*_VEB_ was present in 8.8%, *bla*_GIM_ was present in 15.5%, and *bla*_SIM_ was detected in 6% of the collected strains. Interestingly, while *bla*_OXA-24_ and *bla*_OXA-58_ was not found in this collection, the presence of *bla*_OXA-23_ was detected in 30% of strains and *bla*_OXA-69_ was detected in 15% of the strains (Fig 3C, Table 2).

**Table 2:** PCR based detection of β-lactam resistance determinants.

Strains	β-lactamases							
	Class A		Class B			Class D		
	*bla*_*VEB*_	*bla*_SHV_	*bla*_GIM_	*bla*_IMP_	*bla*_SIM_	*bla*_*oxa 23*_	*bla*_*oxa 58*_	*bla*_*OXA69*_
KPN1	+	+	-	+	-	+	-	+
KPN2	-	+	-	-	-	-	-	-
KPN3	-	+	-	-	-	-	-	-
KPN4	-	-	-	-	+	-	-	-
KPN5	-	-	-	-	-	-	-	-
KPN6	-	-	-	-	-	-	-	+
KPN7	-	-	-	-	-	-	-	-
KPN8	-	-	-	-	-	-	-	-
KPN9	-	+	-	-	-	-	-	-
KPN10	-	+	-	-	+	-	-	-
KPN11	+	+	-	-	+	-	-	+
KPN12	-	-	-	-	-	-	-	-
KPN13	-	-	-	-	-	-	-	-
KPN14	-	-	-	-	-	-	-	-
KPN15	+	-	-	+	-	+	-	+
KPN16	-	-	-	-	-	-	-	-
KPN17	-	-	-	-	-	+	-	-
KPN18	-	-	-	-	-	-	-	-
KPN19	-	-	-	-	-	-	-	-
KPN20	-	-	-	-	-	+	-	-
KPN21	-	-	-	-	-	-	-	-
KPN22	-	-	-	-	-	-	-	-
KPN23	-	-	-	-	-	-	-	-
KPN24	-	+	-	-	-	-	-	-
KPN25	-	-	-	-	-	-	-	-
KPN26	-	-	-	-	-	-	-	-
KPN27	-	-	-	-	-	-	-	-
KPN28	-	+	-	-	-	-	-	-
KPN29	-	-	-	-	-	-	-	-
KPN30	-	-	-	-	-	-	-	-
KPN31	-	-	-	-	-	-	-	-
KPN32	-	-	-	-	-	-	-	-
KPN33	-	-	+	-	-	-	+	-
KPN34	+	-	-	-	-	-	-	+
KPN35	-	-	+	-	-	-	-	-
KPN36	-	-	-	-	-	-	+	+
KPN37	-	-	+	-	-	-	+	-
KPN38	-	-	+	-	-	-	-	-
KPN39	-	-	-	-	-	-	-	-
KPN40	-	-	-	-	-	-	-	-
KPN41	-	-	+	-	-	-	-	-
KPN42	-	-	-	-	-	-	-	-
KPN43	-	-	+	-	-	-	-	-
KPN44	-	-	+	-	-	-	-	-
KPN45	-	-	-	-	-	-	-	+

‘+’ indicates presence of the respective gene in the particular strain; ‘-‘indicates respective gene was not found in the particular strain

- The aminoglycoside adenyl transferase *aadA1* was detected in 30% of the isolates, *aadB* was detected in 3% of these strains, *aphA3* was detected in 17% of the isolates, *aphA6* was detected in 6% of the isolates. The rRNA methyl transferase alleles for conferring resistance to aminoglycosides *e*.*g*.*rmtB* was detected in 11% of the isolates, *rmtC* was detected in 13% of the isolates, and surprisingly *rmtD* was not found in the isolates. Linked genes *strA-strB* present on mobile elements of pathogenic organisms is main contributor for resistance to streptomycin. The presence of *strA* was detected in 20% of these isolates and the presence of *strB* was detected in 13% of these isolates (Fig 3C, Table 3).

**Table 3:** PCR based detection of other resistance determinants.

Strains	Insertion elements		Aminoglycosidase									Quinolones			others		
	*5'cs*	*Class I int*	*aadA1*	*aadB*	*aphA3*	*aphA6*	*rmtB*	*rmtC*	*rmtD*	*strA*	*strB*	*qnrA*	*qnrB*	*qnrS*	*cat III*	*catB2*	*catB3*
KPN1	+	-	+	+	+	+	+	+	-	+	+	-	-	-	-	-	-
KPN2	+	-	+	-	-	-	-	-	-	-	-	+	-	-	-	-	-
KPN3	+	-	+	-	-	-	-	+	-	-	-	-	+	-	-	-	-
KPN4	-	-	-	-	-	-	-	-	-	-	-	-	-	-	-	-	-
KPN5	-	-	-	-	-	-	-	-	-	-	-	-	-	-	-	-	-
KPN6	-	-	-	-	-	+	-	-	-	-	-	-	-	-	-	-	-
KPN7	-	-	-	-	-	-	-	-	-	-	-	-	-	-	-	-	-
KPN8	-	-	-	-	-	-	+	-	-	-	-	+	-	+	-	-	-
KPN9	-	-	-	-	-	-	-	-	-	+	+	-	+	-	-	-	-
KPN10	-	-	-	-	-	-	-	-	-	-	-	-	-	-	-	-	-
KPN11	+	-	+	-	-	+	+	-	-	+	+	-	-	-	-	-	-
KPN12	-	-	-	-	-	-	-	+	-	-	-	-	-	-	-	-	-
KPN13	-	-	-	-	-	-	-	-	-	-	-	-	-	-	-	-	-
KPN14	+	-	+	-	+	-	-	-	-	+	-	-	-	-	-	+	-
KPN15	+	-	+	-	+	+	+	-	-	+	+	-	+	-	-	-	-
KPN16	-	-	-	-	-	-	-	-	-	-	-	+	-	-	-	-	-
KPN17	+	-	+	-	-	+	-	-	-	-	-	-	-	-	-	+	-
KPN18	+	-	-	-	-	-	-	-	-	-	-	-	-	-	-	-	-
KPN19	-	-	+	-	-	-	-	+	-	-	-	-	-	-	-	-	-
KPN20	+	-	+	-	-	-	-	-	-	-	-	-	-	-	-	-	-
KPN21	-	-	-	-	-	-	-	-	-	-	-	-	-	-	-	-	-
KPN22	-	-	-	-	-	-	-	-	-	-	-	-	-	-	-	-	-
KPN23	+	-	-	-	-	-	-	-	-	-	-	-	-	+	-	-	-
KPN24	-	-	-	-	+	-	-	-	-	+	-	-	+	-	-	-	-
KPN25	-	-	-	-	-	-	-	-	-	-	-	-	-	-	-	-	-
KPN26	-	-	-	-	-	-	-	-	-	-	-	-	-	+	-	-	-
KPN27	-	+	-	-	-	-	-	-	-	-	-	-	-	-	-	-	-
KPN28	+	-	-	-	+	-	-	-	-	-	-	-	+	-	-	-	-
KPN29	+	-	-	-	+	-	-	-	-	-	-	-	+	-	-	-	-
KPN30	-	-	-	-	-	-	-	-	-	-	-	-	-	+	-	-	-
KPN31	-	+	-	-	-	-	-	-	-	-	-	-	-	-	-	-	-
KPN32	-	+	-	-	-	-	-	-	-	-	-	-	-	-	-	-	-
KPN33	-	+	-	-	-	-	-	-	-	-	-	-	+	-	-	-	-
KPN34	-	+	-	-	+	+	-	+	-	-	-	-	-	-	-	+	-
KPN35	-	+	-	-	+	-	+	-	-	-	-	-	-	-	-	-	-
KPN36	-	+	-	-	-	-	-	-	-	-	-	-	-	-	-	-	-
KPN37	-	+	-	-	+	-	-	+	-	-	-	-	-	-	-	+	-
KPN38	-	+	-	-	-	-	-	-	-	-	-	-	-	-	-	-	-
KPN39	-	+	-	-	-	-	-	-	-	-	-	-	-	-	-	-	-
KPN40	-	+	-	-	-	-	-	-	-	-	-	-	-	-	-	-	-
KPN41	-	+	-	-	-	-	-	-	-	-	-	-	-	-	-	-	-
KPN42	-	+	-	-	-	-	-	-	-	-	-	-	-	-	-	-	-
KPN43	-	+	-	-	+	-	-	-	-	-	-	-	+	-	-	-	-
KPN44	-	+	-	-	+	-	-	-	-	-	-	-	-	-	-	+	-
KPN45	-	+	-	-	-	+	-	-	-	-	-	-	+	-	-	-	-

‘+’ indicates presence of the respective gene in the particular strain; ‘-‘indicates respective gene was not found in the particular strain.

- The presence of *qnr* alleles contributes to the plasmid mediated resistance against quinolone group of antibiotics. The presence of *qnrA* was detected in 10% of the isolates. The presence of *qnrB* was detected in 20% and presence of *qnrS* was detected in 8.8% of the isolates (Fig 3C, Table 3).

### MIC for structurally unrelated compounds

**Fig 4:**
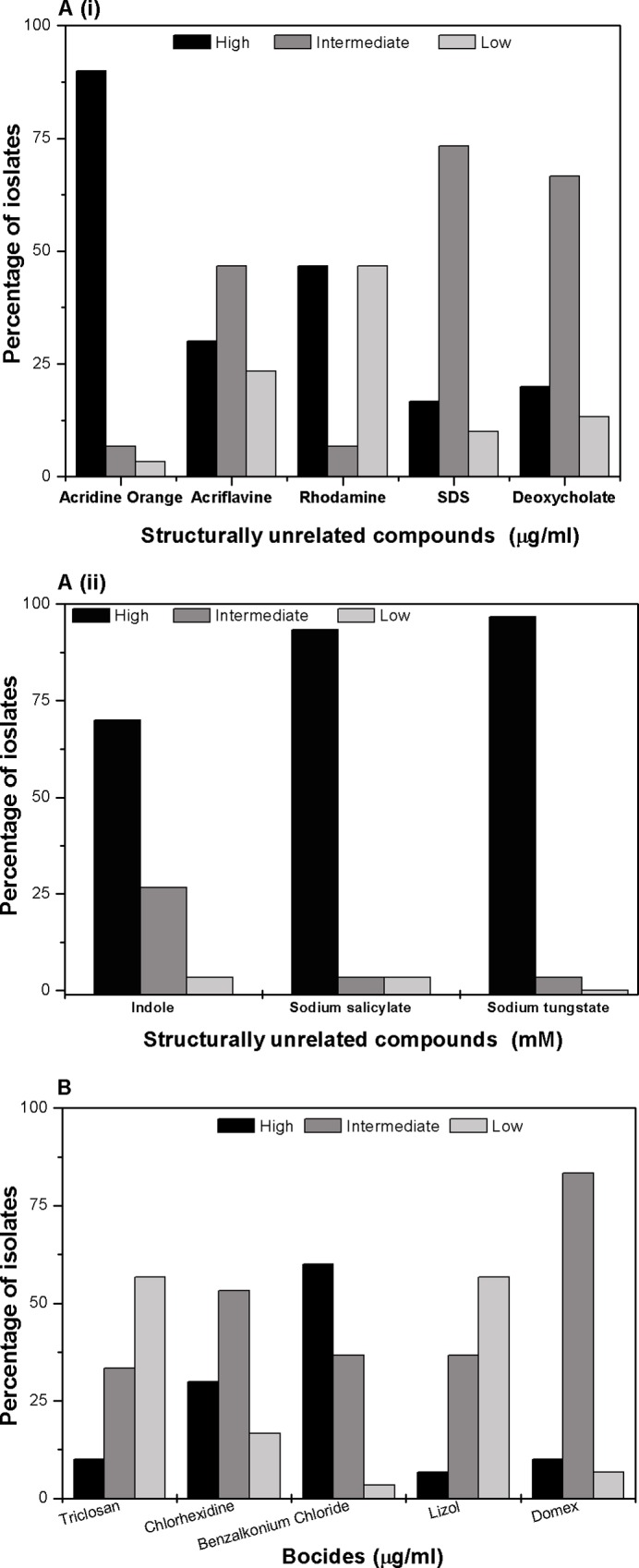
Resistance to SUCs and hospital based disinfectants. (A) i) Percentage of isolates with different level of resistance to different SUCs is shown. Percentage of strains having different MIC values for ACR: acriflavine, AO: acridine orange, RHO: rhodamine, SAF: safranin, EtBr: ethidium bromide, SDS: Sodium dodecyl sulphate and deoxycholate are plotted here. ii) Percentage of strains having different MIC values for IND: indole, SS: sodium salicylate and ST: sodium tungstate is also shown here. The bar graph represents the mean of three independent experiments. (B) Graphical representation of percentage of isolates with different level of resistance with different biocides. In this study, we grouped the isolates in three different resistance level for each biocides viz. strains with high MIC values are grouped as highly resistant, intermediate MIC value as intermediately resistant and low MIC value as sensitive. For this study, we used the following biocides: TRI: triclosan (high MIC value– 0.5 μg/ml or more, intermediate MIC value- 0.1 μg/ml to 0.05 μg/ml and low MIC value- 0.01 μg/ml or less); CHX: chlorhexidine (high MIC value 51.2µg/ml to >102.4 μg/ml, intermediate MIC value 12.8 μg/ml to 25.6 μg/ml and low MIC value3.2 μg/ml or less); BZC: benzalkonium chloride (MIC values similar as CHX); lizol (high MIC value 1.2% to 1.6%, intermediate MIC value 0.4% to 0.8% and low MIC value 0.2% or less); and domex (MIC values similar as lyzol). The bar graph represents the mean of three independent experiments.

MIC for different SUCs was determined and classification was made based on their extent of resistance against different SUCs. For acriflavine, 30% of the isolates were intermediately resistant showing MIC values of 256 μg/ml to 1024 μg/ml and other 46.6% of isolates are less resistant having MIC values in the range of 64 μg/ml or less. For acridine orange a dye (90%) of the isolates were intermediately resistant showing MIC values of between 256 μg/ml to 1024 μg/ml. For rhodamine G another dye 46% of the isolates were highly resistant showing MIC values of 4096 μg/ml to 16384 μg/ml or more and 6% of isolates have MIC values 1024 μg/ml to 256 μg/ml and 46% of isolates are less resistant having MIC values in the range of 64 μg/ml to 16 μg/ml or less. For safranin 9% of the isolates were highly resistant showing MIC values of 4096 μg/ml to 16384 μg/ml or more and 85% of isolates have MIC values 1024 μg/ml to 256 μg/ml and 6.5% of isolates are less resistant having MIC values in the range of 64 μg/ml to 16 μg/ml or less. For ethidium bromide another dye 83% of isolates have MIC values 1024 μg/ml to 256 μg/ml and remaining 15% of isolates are less resistant having MIC values in the range of 64 μg/ml to 16 μg/ml or less. For sodium dodecyl sulphate a detergent 16% of the isolates were highly resistant showing MIC values of 4096 μg/ml to 16384 μg/ml or more and 73% of isolates have MIC values 1024 μg/ml to 256 μg/ml and 10% of isolates are susceptible having MIC values in the range of 64 μg/ml to 16 μg/ml or less. For deoxycholate a bile salt 20% of the isolates were highly resistant showing MIC values of 4096 μg/ml to 16384 μg/ml or more and 67% of isolates have MIC values 1024 μg/ml to 256 μg/ml and 13% of isolates are susceptible having MIC values in the range of 64 μg/ml to 16 μg/ml or less (Fig 4A, i).

For indole 70% of the isolates were intermediately resistant showing MIC values of 2.5 mM and 27% of isolates are susceptible having MIC values in the range of 1mM or less. For sodium salicylate 94% of the isolates were highly resistant showing MIC values of 10 mM to 25 mM or more and 3% remaining isolates are sensitive having MIC of 1 mM. For sodium tungstate 97% of the isolates were highly resistant showing MIC values of more than 25 mM (Fig 4A, ii).

### Susceptibility towards hospital based biocides

Apart from antibiotics used for treatment, bacteria were found resistant to biocides which are regularly used in hospital as cleansing materials which led them stay on clinical set up for long and thus making patients vulnerable to nosocomial infections. For benzalkonium chloride, 60% of the isolates showed high MIC values ranging from 51.2 μg/ml to >102.4 μg/ml and another 36% of isolates were found intermediately resistant having MIC values in the range of 12.8 μg/ml 25.6 μg/ml and only 3% of strain showed lower MIC values of 3.2 μg/ml or less. For chlorhexidine 30% of the isolates showed high MIC values of 25.6 μg/ml and another 53% of isolates were intermediately resistant having MIC values in the range of 6.4 μg/ml to 12.5 μg/ml and 16% of strain showed lower MIC values of 3.2 or less. For triclosan more than half (56%) of the isolates were susceptible showing MIC values for 0.01% solution and 10% of isolates had MIC values 0.5% solution and another 33% of isolates are intermediately resistant having MIC values in the range of 0.1% to 0.05% solution.

For Domex a commercially available disinfectant 83% of isolates shown MIC of 0.4 μg/ml to 0.8 μg/ml i.e. and also 10% of them have MIC of 1.6 μg/ml or more and 6% of the isolates were susceptible showing MIC values of <0.1 μg/ml. For lizol another commercially available disinfectant 36% of isolates shown MIC of 0.4% to 0.8% of the solution i.e. and also 6% of them have MIC of 1.2% to 1.6% solution or more and 56% of the isolates were susceptible showing MIC values of 0.2% to <0.1% solution (Fig 4B).

Overall we found the clinical isolates in our collection were not only multidrug resistant but were also broadly biocide tolerant.

### Role of Efflux activity in Antimicrobial resistance

**Fig 5:**
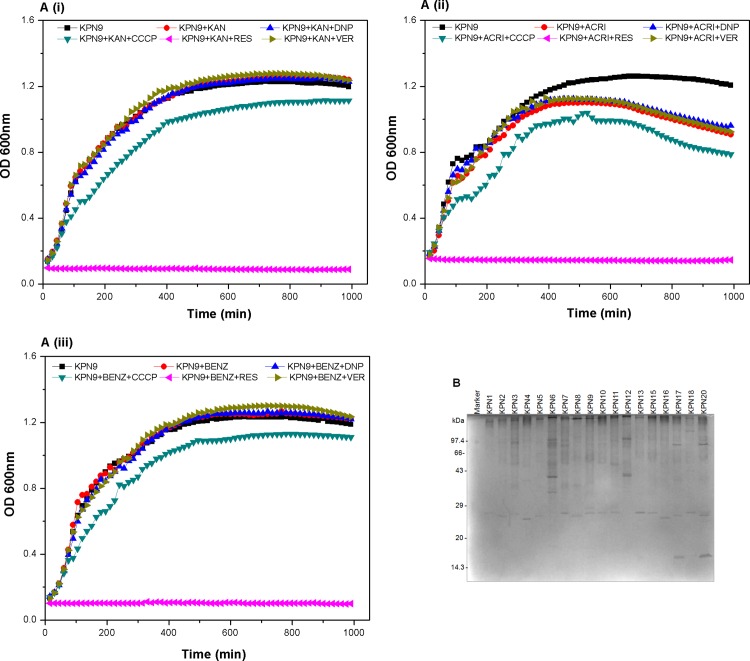
Growth inactivation assay and OMP profiles in *K*. *pneumoniae* strains. (A) Graphical presentation of growth inactivation assay using *K*. *pneumoniae* strain with different antimicrobial compounds and efflux pump inhibitors is shown by OD_600nm_ at every 15 minutes time interval. Growth inactivation assays (GIAs) were done with a representative strain to know the impact on drug efflux capability using substrates KAN: kanamycin, ACRI: acriflavine and BENZ: benzalkonium chloride, in presence and absence of efflux pump inhibitors (CCCP: carbonyl cyanide 3- chlorophenylhydrazone, DNP: 2, 4 -dinitrophenol, VER: verapamil, and RES: reserpine). The graph represents the mean of three independent experiments. (B) Protein gel picture of OMP profile of representative *K*. *pneumoniae strai*ns. OMP was isolated from the strains using sarkosyl method and was checked on SDS-PAGE. *K*. *pneumoniae* isolates showed differential expression in our collection.

In our study, to investigate the role of efflux system in drug resistance we performed growth inactivation assays with different antimicrobials and inhibitors which act by hindering active efflux activity. It was interesting to see that the MDR strain when grown in kanamycin as a substrate without the inhibitor, after >12 hrs of growth, showed 1.19—fold, 1.01—fold, 0.98—fold and 11.83—fold increased growth when compared to its growth in presence of inhibitors CCCP, DNP, verampamil and reserpine respectively in independent experiments (Fig 5A, i).

When MDR strain was grown in acriflavine as a substrate without the inhibitor, after >12 hrs of growth, showed 1.13—fold, 0.96—fold, 0.97—fold and 6.4—fold increased growth when compared to its growth in presence of inhibitors CCCP, DNP, verampamil and reserpine respectively in independent experiments (Fig 5A, ii). When MDR strain was grown in benzalkonium chloride as a substrate without the inhibitor, after >12 hrs of growth, showed 1.16—fold, 1.01—fold, 0.99—fold and 10.44—fold increased growth when compared to its growth in presence of inhibitors CCCP, DNP, verampamil and reserpine respectively in independent experiments (Fig 5A, iii). Overall, we found the clinical isolates in our collection use active efflux as the underlying molecular mechanism to mediate not only multidrug resistance but also biocide tolerance.

### Outer membrane protein profiles of Indian *K*. *pneumoniae* isolates

The cell envelope is the prime line for most outside stress conditions that may modify envelope components and thus bring an extra cytoplasmic stress response. A reduction in the permeation of antibiotics is generally related to a decrease in porin expression or an alteration in the porin structure. The role of outer membrane proteins were also analysed among the clinical isolates. The outer membrane proteins were isolated and checked on SDS- PAGE. Differential patterns of outer membrane proteins amongst the clinical strains could be observed and the proteins over expressed bands from multidrug resistant strains were identified. The over expressed bands may be part of efflux pumps, so we are now working towards the identification of those interestingly distinguished protein (Fig 5B).

### Determination of presence of CusABC efflux pumps

With the proof of efflux activity through efflux pumps in the representative strains it is necessary to know the presence of efflux pumps. Therefore the genomic DNA of the isolates and gene specific primers were used to amplify the AcrAB-like CusABC efflux genes. Among the pool of strains used in this study 51% of strains were having RND efflux genes, while 33% percent of the strains also showed the presence of unique regulator *cue*R gene (data not shown), work are in progress to elucidate and delineate their impact in drug resistance in *K*. *pneumoniae*.

### Oxidative stress behavior of Indian *K*. *pneumoniae* isolates

**Fig 6:**
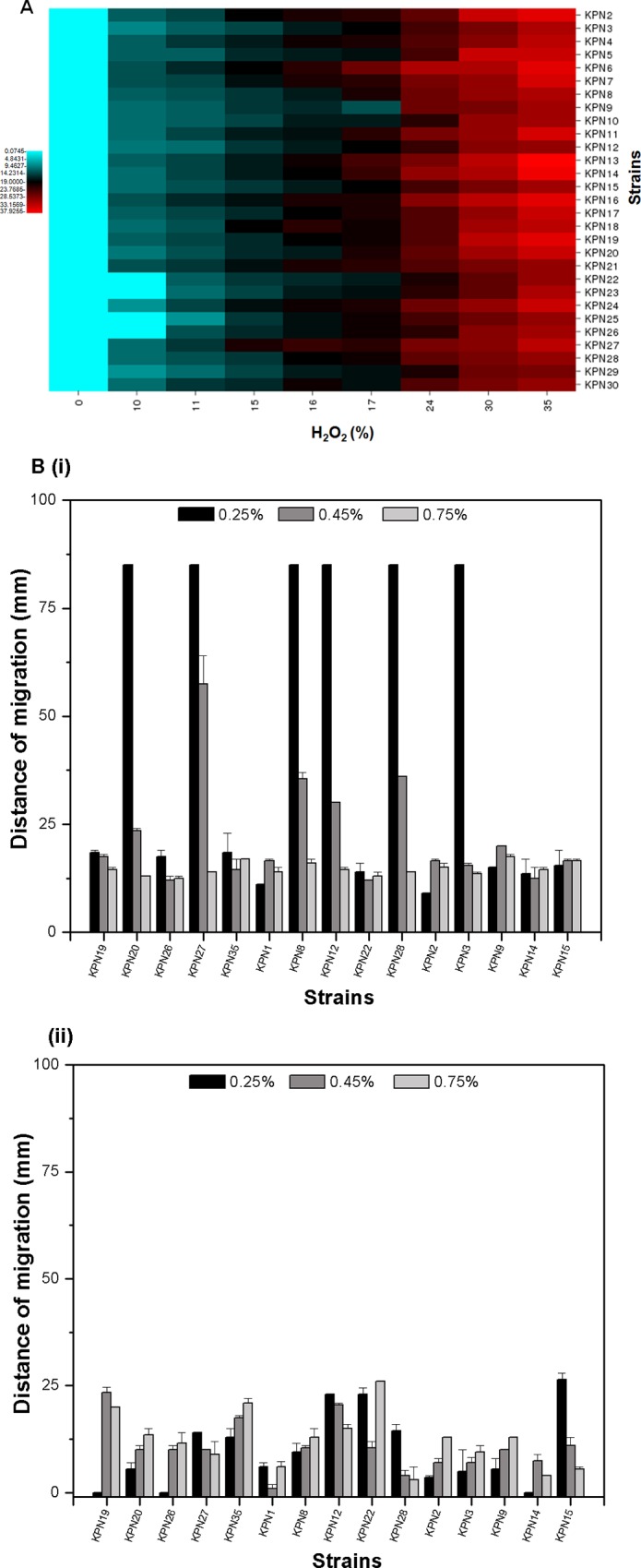
Oxidative stress and bioflim formating assay in *K*. *pneumoniae* strains. (A) Diagram showing the zone of inhibition in different strains due to the different concentration of H_2_O_2._ In this study 0%, 0.1%, 1%, 3%, and 10% of H_2_O_2_ were used as oxidative stress inducing agent. The diagram represents the mean of three independent experiments. (B) Biofilm formation by the *K*. *pneumoniae* isolates are shown here: i) tubes showing stained biofilm rings in few representative isolates ii) graphical presentation of biofilm formation among the isolates is shown as the ratio of OD_570nm_ and OD_600nm_. The bar graph represents the mean of three independent experiments.

We performed oxidative disc diffusion assay to know the oxidative stress tolerance of the clinical isolates. Hydrogen peroxide (H_2_O_2_) was used to generate oxidative stress in our current study. It was found that 3.3% of strains were resistant (diameter of zone of inhibition with 1% H_2_O_2_ is 8 mm or less) toward oxidative stress and another 53% were intermediately resistant (diameter of zone of inhibition with 1% H_2_O_2_ is 8 mm– 13 mm) and the other 43.3% were less susceptible (diameter of zone of inhibition with 1% H_2_O_2_ is 14 mm or more) (Fig 6A).

### Motility and Biofilm formation by Indian *K*. *pneumoniae* isolates

**Fig 7:**
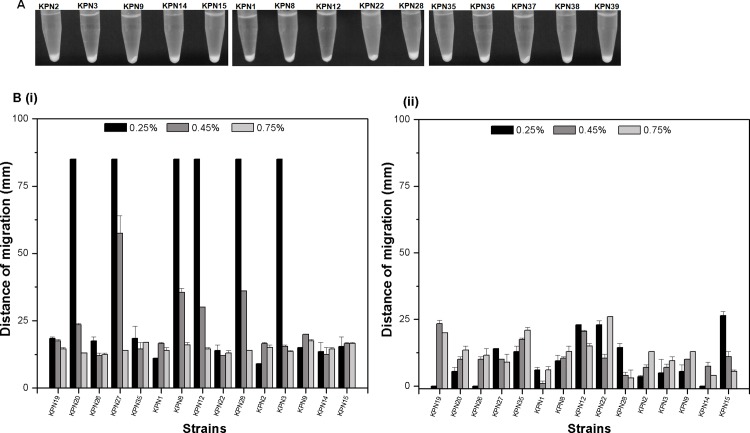
Precipitation and motility profile of *K*. *pneumoniae* isolates obtained in medical centre in India. (A) Tubes showing precipitation of culture in our collection of isolates. None of the isolates formed a mucoidy supernatant, pellet formed was dense. (B) The motility behavior shown by the different *K*. *pneumoniae* isolates were shown; we carried out motility assay on soft agar (0.25%, 0.45% and 0.7% agar) and measured as zone of migration (mm) from the point of inoculation; i) graphical representation of swarming motility in *K*. *pneumoniae* isolates shown as zone of migration of isolates on surface of the media, ii) graphical representation of twitching motility in *K*. *pneumoniae* isolates shown as zone of migration of isolates under the media. The bar graph represents the mean of three independent experiments.

We performed the biofilm assay using LB broth medium in glass tubes. From this study it is found that most of the strains formed biofilm (Fig 6B, i) and upon comparing with their antibiogram it was found that > 80% of the strains that had an ability to form strong biofilms were resistant (Fig 6B, ii). Strains were tested for their ability to form dense pellet by centrifugation, and none were found to form loose pellet, all strains formed dense pellet (Fig 7A). We performed motility assay to check for swarming motility (Fig 7B, i) and twitching motility (Fig 7B, ii) among 15 representative isolates using soft agar plates of 0.25% agar, 0.45% agar and 0.7% agar. In this study KPN23, KPN27, KPN8, KPN12, KPN28, KPN3 isolates showed maximum swarming motility with >60 mm of zone of motility at 0.25% agar concentration.

## Discussion

Over the millennia, bacteria have evolved evasion strategies to overcome arrays of chemical and environmental assails including antimicrobial drugs [1]. The occurrence of nosocomial infections in hospital intensive care units due to *K*. *pneumoniae* currently ranges from 2% to 10% of all Gram-negative bacterial infections in Europe and account for about 2.5% of them in the United States [2]. *K*. *pneumoniae* is a rapidly emerging nosocomial pathogen that causes wide variety of infections from mild to severe including bacteremia, pneumonia, meningitis, urinary tract infections, wound infections and neonatal septicemia [1]. Infectious disease society of America has reported this organism to be amongst top five causing major diseases and mortality in the United States [5]. It has now become a major cause of hospital-acquired infections worldwide due to its remarkable propensity to rapidly acquire resistance determinants to a wide range of antibacterial agents [6]. It has been included in the acronym ESKAPE which lists top six most notorious Gram negative bacteria [7]. The capacity of this pathogen to cause major outbreaks stems from two major characteristics; first one is antimicrobial resistance and second one being the ability to persist in the environment for extended periods of time [22]. There has been no significant study till date that describes the variation in the clinical strains that are isolated and mechanisms of drug resistance displayed by them. Therefore, we performed this longitudinal study, in the present study we collected multiple clinical strains of *K*. *pneumoniae* and started from their clonal studies and after knowing that they belonged to distinct clonal classes we worked further.

We found the strains to be multidrug resistant as determined by Kirby Bauer disc diffusion method and their MIC values. We screened for the presence of various resistance determinants by PCR method. Like any Gram-negative bacteria, our isolates also harbored multiple β-lactamases which explains their resistant phenotype. It is generally considered that clinical strains contain integrons because of the multi-drug resistance advantages that these mobile DNA elements facilitate [32]. Though class 1 integron was not found in our study, multiple genes (the AME’s and *aphA6*) encoding resistance to clinically relevant antimicrobials [32], particularly aminoglycosides were found which indicate that the strains could have evolved with them to circumvent the regime of drug assault such as amikacin, gentamicin and streptomycin.

Studies on the mechanisms of quinolone resistance in Gram-negative bacteria such as *E*. *coli*, *N*.*gonorrhoeae*, *V*. *cholerae* and *Haemophilus influenzae* have elucidated that the alterations in the GyrA subunit of DNA gyrase are primarily responsible for the high level resistance to nalidixic acid [32]. In *E*. *coli*, mutations are clustered in the quinolone resistance-determining region (QRDR) located between amino acids Ala-67 andGln-106, the most frequent being at codon 83 or at equivalent positions in other microorganisms [43]. Several reports have discussed the significance of alteration in ParC subunit of topoisomerase IV in enhancing resistance to fluoroquinolones. However we were unable to identify any mutations in the previously defined QRDR in our collection of nalidixic acid resistant isolates respectively. As efflux pumps are one of the main contributors to drug resistance, we screened for the presence of some efflux pumps by PCR and also established the activity of efflux pumps by performing the growth inactivation assay.

We also determined the resistance to biocides as they are the major surface elements to which the clinical strains are exposed to. So this study again proves that efflux pumps are not only conferring drug resistance but also mediates resistance to a variety of structurally unrelated compounds which led to therapy failure by increasing the MIC of antimicrobials. This active drug transport is involved in low intrinsic susceptibility, cross-resistance to chemically unrelated classes of molecules, and selection/acquisition of additional mechanisms of resistance. We were also able to detect the presence of protein kinases in these strains, and currently our research focuses on their biological relevance.

The goal of this study was to link the phenotypes and the resistance determinants in *K*. *pneumoniae*, giving a picture of the complex endemic nature in this geographic location. From these data, it is clear that the potential impact of *K*. *pneumoniae* infections on humans in Indian hospitals is significant. Taken together, prevalence of resistance determinants for the front line choice of drugs threatens the future continued clinical use of antibiotics. Therefore, the breadth of resistome, monotony of resistance mechanisms, evolution and emergence of antibiotic resistant populations needs to be clearly understood to allow the discovery of new therapeutic drugs.
