# Correction: Occurrence of Diverse Antimicrobial Resistance Determinants in Genetically Unrelated Biocide Tolerant *Klebsiella pneumoniae*

**DOI:** 10.1371/journal.pone.0175487

**Published:** 2017-04-05

**Authors:** Amitabha Mondal, Manjunath Venkataramaiah, Govindan Rajamohan, Vijaya Bharathi Srinivasan

In Fig 6, images B (i) and B (ii) are incorrectly displayed. Please see the corrected Fig 6 here.

**Fig 6:**
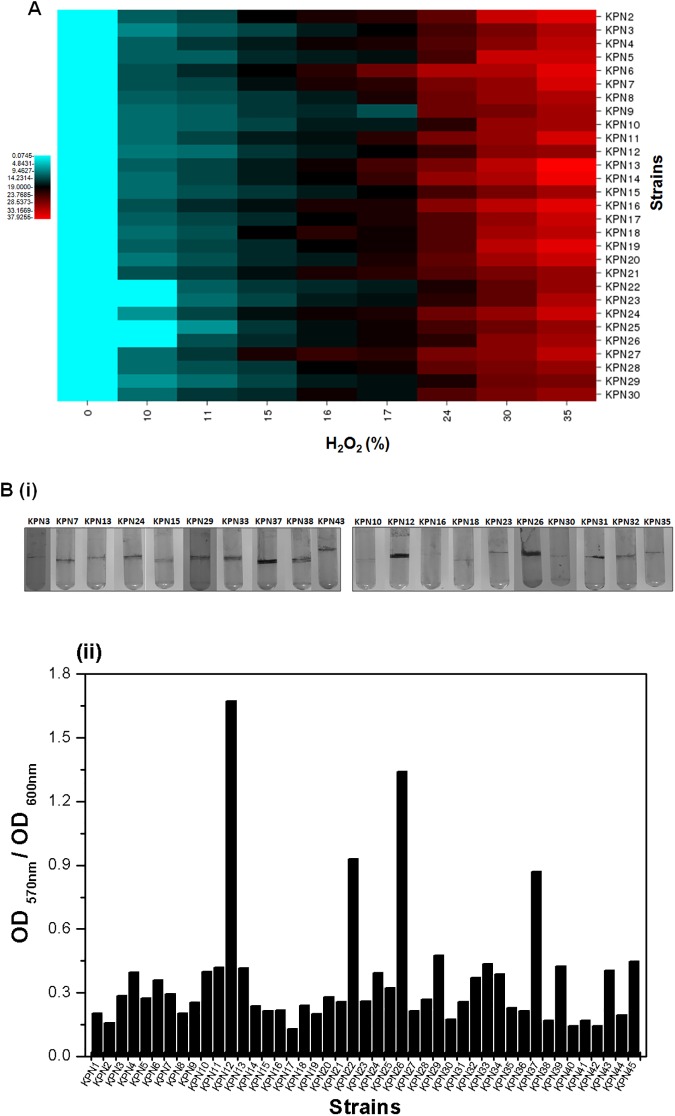
Oxidative stress and bioflim formating assay in *K*. *pneumoniae* strains. (A) Diagram showing the zone of inhibition in different strains due to the different concentration of H_2_O_2_. In this study 0%, 0.1%, 1%, 3%, and 10% of H_2_O_2_ were used as oxidative stress inducing agent. The diagram represents the mean of three independent experiments. (B) Biofilm formation by the *K*. *pneumoniae* isolates are shown here: i) tubes showing stained biofilm rings in few representative isolates ii) graphical presentation of biofilm formation among the isolates is shown as the ratio of OD_570nm_ and OD_600nm_. The bar graph represents the mean of three independent experiments.
